# Crystal structure of ethyl 3-amino-6-methyl-2-[(4-methyl­phen­yl)carbamo­yl]-4-[(*E*)-2-phenyl­ethen­yl]thieno[2,3-*b*]pyridine-5-carboxyl­ate monohydrate

**DOI:** 10.1107/S2056989016001341

**Published:** 2016-02-06

**Authors:** Joel T. Mague, Mehmet Akkurt, Shaaban K. Mohamed, Etify A. Bakhite, Mustafa R. Albayati

**Affiliations:** a Department of Chemistry, Tulane University, New Orleans, LA 70118, USA; b Department of Physics, Faculty of Sciences, Erciyes University, 38039 Kayseri, Turkey; c Chemistry and Environmental Division, Manchester Metropolitan University, Manchester M1 5GD, England; d Chemistry Department, Faculty of Science, Minia University, 61519 El-Minia, Egypt; e Chemistry Department, Faculty of Science, Assiut University, Assiut 71516, Egypt; f Kirkuk University, College of Science, Department of Chemistry, Kirkuk, Iraq

**Keywords:** crystal structure, thienyl ring, pyridine ring, dimer, *PLATON* SQUEEZE

## Abstract

In the title mol­ecule, C_27_H_25_N_3_O_3_S·H_2_O, the dihedral angle between the planes of the thienyl ring and the pendant *p*-tolyl group is 39.25 (6)°, while that between the pyridine ring and the pendant phenyl ring is 44.37 (6)°. In addition, there is a slight twist in the bicyclic core, with a dihedral angle of 2.39 (4)° between the thienyl and pyridine rings. The conformation of the carbamoyl moiety is partially determined by an intra­molecular N—H⋯O hydrogen bond. In the crystal, complementary N—H⋯O hydrogen bonds form dimers which are then associated into chains parallel to the *c* axis through O—H⋯N hydrogen bonds involving the water mol­ecule of crystallization. Electron density associated with an additional solvent mol­ecule of partial occupancy and disordered about a twofold axis was removed with the SQUEEZE procedure in *PLATON* [Spek (2015 ▸). *Acta Cryst.* C**71**, 9–18]. The given chemical formula and other crystal data do not take into account the unknown solvent molecule(s).

## Abstract 2

In the crystal, complementary N—H⋯O hydrogen bonds form dimers which are then associated into chains parallel to the *c* axis through O—H⋯N hydrogen bonds involving the lattice water mol­ecule.

## Chemical context   

Recently, considerable inter­est has been focused on the synthesis and pharmacological activities of thieno[2,3-*b*]pyridine derivatives (Bakhite, 2003 ▸). They are versatile synthons such that a variety of new heterocycles with good pharmaceutical profiles can be designed (Litvinov *et al.*, 2005 ▸). These thieno[2,3-*b*]pyridines are usually prepared through *S*-alkyl­ation of 3-cyano­pyridine-2(1*H*)-thio­nes and subsequent Thorpe–Ziegler isomerization of the resulting 2-(alkyl­thio)­pyridine-3-carbo­nitriles (Litvinov *et al.*, 2005 ▸). On the other hand, a literature survey revealed that only a few 4-(2-phenyl­ethyl­ene)thieno[2,3-*b*]pyridines, without any X-ray diffraction analyses, have been reported (Ho & Wang, 1995 ▸). The above findings promoted us to synthesize the title compound and characterize its crystal structure.
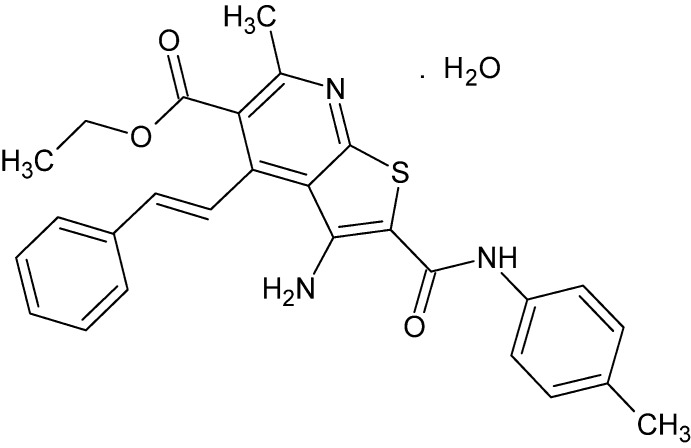


## Structural commentary   

**Figure 1:**
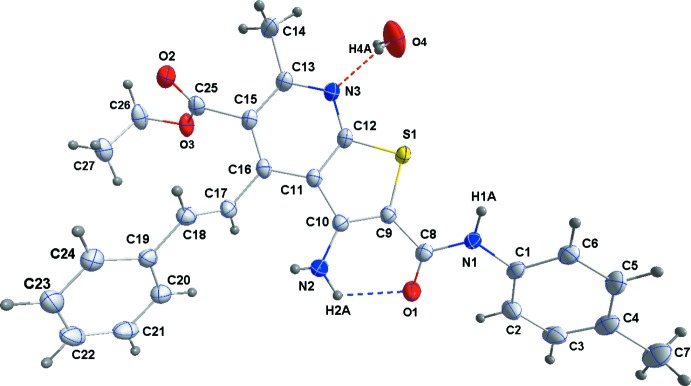
The mol­ecular structure of the title compound, shown with 50% probability ellipsoids. Hydrogen bonds are shown by dotted lines.

In the title mol­ecule, the dihedral angle between the thienyl ring and the pendant *p*-tolyl group is 39.25 (6)° while that between the pyridine ring and the pendant phenyl ring is 44.37 (6)°. In addition there is a slight twist in the bicyclic core with a dihedral angle of 2.39 (4)° between the thienyl and pyridine rings. The conformation of the carbamoyl moiety is partially determined by an intra­molecular N2—H2*A*⋯O1 hydrogen bond (Table 1 ▸ and Fig. 1 ▸).

## Supra­molecular features   

**Figure 2:**
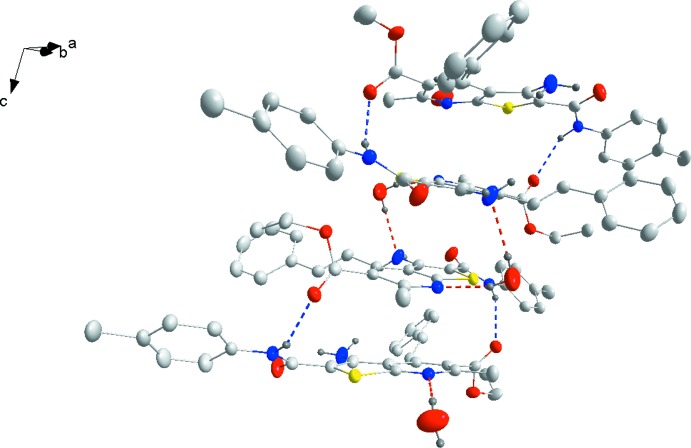
View of the hydrogen-bonded dimer with half of each of two adjacent dimers as the basic elements of the one-dimensional chains. Dashed lines indicate hydrogen bonds. H atoms not involved in hydrogen bonding have been omitted. Displacement ellipsoids are drawn at the 50% probability level.

In the crystal, complementary N1—H1*A*⋯O2^i^ [symmetry code: (i) 1 − *x*, *y*, 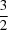 − *z*] form dimers which are then associated into chains parallel to the *c* axis through O4—H4*A⋯*N3 and O4—H4*B*⋯N2^ii^ [symmetry code: (ii) 1 − *x*, 1 − *y*, 1 − *z*] hydrogen bonds involving the water mol­ecules of crystallization (Fig. 2 ▸ and Table 1 ▸).

## Synthesis and crystallization   

The title compound was prepared by heating equimolar qu­anti­ties of ethyl 3-cyano-1,2-di­hydro-6-methyl-4-(2-phenyl­ethen­yl)-2-thioxo­pyridine-5-carboxyl­ate and chloro­(*N*-(4-methyl­phen­yl)acetamide (10 mmol) in absolute ethanol (25 ml) containing sodium ethoxide (0.3 g) on a steam bath for 30 mins. The product that formed on cooling was collected by filtration and recrystallized from ethanol 95% as yellow needles. Yield (73%); m.p. IR (KBr) ν = 3500, 3350, (NH_2_, NH), 1701 (C=O, ester), 1638 (C=O, amide) cm^−1. 1^H NMR (DMSO-*d*_6_): 9.41 (*s*, 1H, NH), 7.73–7.75 (*d*, *J* = 16 Hz, 1H, ethene proton), 7.64–7.66 (*d*, *J* = 16 Hz, 2H, ArH), 7.55–7.56 (*d*, *J* = 8 Hz, 2H, ArH), 7.38–7.44 (*m*, 3H, ArH), 7.13–7.15 (*d*, *J* = 16 Hz, 2H, ArH), 6.81–6.85 (*d*, *J* = 16 Hz, 1H, ethene proton).

## Refinement   

Crystal data, data collection and structure refinement details are summarized in Table 2 ▸. C-bound H atoms were placed in calculated positions (C—H = 0.95–0.99 Å) while those attached to N or O atoms were placed in locations derived from a difference Fourier map and their coordinates adjusted to give N—H = 0.91 and O—H = 0.85 Å. All were included as riding contributions with isotropic displacement parameters 1.2–1.5 times those of the attached atoms. Electron density associated with an additional solvent mol­ecule of partial occupancy and disordered about a twofold axis was removed with the SQUEEZE procedure in *PLATON* (Spek, 2015 ▸).

## Supplementary Material

Crystal structure: contains datablock(s) global, I. DOI: 10.1107/S2056989016001341/rz5183sup1.cif

Structure factors: contains datablock(s) I. DOI: 10.1107/S2056989016001341/rz5183Isup2.hkl

Supporting information file. DOI: 10.1107/S2056989016001341/rz5183Isup3.cml

CCDC reference: 1448789

Additional supporting information:  crystallographic information; 3D view; checkCIF report

## Figures and Tables

**Table 1:** Hydrogen-bond geometry (Å, °)

*D*—H⋯*A*	*D*—H	H⋯*A*	*D*⋯*A*	*D*—H⋯*A*
N1—H1*A*⋯O2^i^	0.91	2.17	2.9900 (17)	149
N2—H2*A*⋯O1	0.91	2.25	2.820 (2)	120
O4—H4*A*⋯N3	0.85	2.04	2.863 (2)	163
O4—H4*B*⋯N2^ii^	0.85	2.17	2.967 (2)	157

Symmetry codes: (i) 
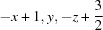
; (ii) 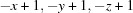.

**Table 2:** Experimental details

Crystal data	
Chemical formula	C_27_H_25_N_3_O_3_S·H_2_O
*M*_r_	489.57
Crystal system, space group	Monoclinic, *C*2/*c*
Temperature (K)	150
*a*, *b*, *c* (Å)	31.083 (3), 12.0766 (10), 14.7678 (12)
β (°)	109.446 (1)
*V* (Å^3^)	5227.2 (7)
*Z*	8
Radiation type	Mo *K*α
μ (mm^−1^)	0.16
Crystal size (mm)	0.28 × 0.15 × 0.10

Data collection	
Diffractometer	Bruker SMART APEX CCD
Absorption correction	Multi-scan (*SADABS*; Bruker, 2015 ▸)
*T*_min_, *T*_max_	0.86, 0.98
No. of measured, independent and observed [*I* > 2σ(*I*)] reflections	24570, 6682, 4746
*R*_int_	0.036
(sin θ/λ)_max_ (Å^−1^)	0.682

Refinement	
*R*[*F* ^2^ > 2σ(*F* ^2^)], *wR*(*F* ^2^), *S*	0.047, 0.140, 1.08
No. of reflections	6682
No. of parameters	319
H-atom treatment	H-atom parameters constrained
Δρ_max_, Δρ_min_ (e Å^−3^)	0.56, −0.65

Computer programs: *APEX2* and *SAINT* (Bruker, 2015 ▸), *SHELXT* (Sheldrick, 2015*a* ▸), *SHELXL2014* (Sheldrick, 2015*b* ▸), *SHELXTL* (Sheldrick, 2008 ▸) and *DIAMOND* (Brandenburg & Putz, 2012 ▸).

## supplementary crystallographic information

### Crystal data

**Table:**

C_27_H_25_N_3_O_3_S·H_2_O	*F*(000) = 2064
*M**_r_* = 489.57	*D*_x_ = 1.244 Mg m^−^^3^
Monoclinic, *C*2/*c*	Mo *K*α radiation, λ = 0.71073 Å
*a* = 31.083 (3) Å	Cell parameters from 7720 reflections
*b* = 12.0766 (10) Å	θ = 2.2–28.7°
*c* = 14.7678 (12) Å	µ = 0.16 mm^−^^1^
β = 109.446 (1)°	*T* = 150 K
*V* = 5227.2 (7) Å^3^	Column, yellow
*Z* = 8	0.28 × 0.15 × 0.10 mm

### Data collection

**Table:**

Bruker SMART APEX CCD diffractometer	6682 independent reflections
Radiation source: fine-focus sealed tube	4746 reflections with *I* > 2σ(*I*)
Graphite monochromator	*R*_int_ = 0.036
Detector resolution: 8.3333 pixels mm^-1^	θ_max_ = 29.0°, θ_min_ = 1.4°
φ and ω scans	*h* = −41→42
Absorption correction: multi-scan (*SADABS*; Bruker, 2015)	*k* = −16→16
*T*_min_ = 0.86, *T*_max_ = 0.98	*l* = −20→20
24570 measured reflections	

### Refinement

**Table:**

Refinement on *F*^2^	Primary atom site location: structure-invariant direct methods
Least-squares matrix: full	Secondary atom site location: difference Fourier map
*R*[*F*^2^ > 2σ(*F*^2^)] = 0.047	Hydrogen site location: mixed
*wR*(*F*^2^) = 0.140	H-atom parameters constrained
*S* = 1.08	*w* = 1/[σ^2^(*F*_o_^2^) + (0.0777*P*)^2^ + 0.6476*P*] where *P* = (*F*_o_^2^ + 2*F*_c_^2^)/3
6682 reflections	(Δ/σ)_max_ = 0.001
319 parameters	Δρ_max_ = 0.56 e Å^−^^3^
0 restraints	Δρ_min_ = −0.65 e Å^−^^3^

### Special details

**Table:**

Experimental. The diffraction data were collected in three sets of 363 frames (0.5° width in ω) at φ = 0, 120 and 240°. A scan time of 80 sec/frame was used.
Geometry. All e.s.d.'s (except the e.s.d. in the dihedral angle between two l.s. planes) are estimated using the full covariance matrix. The cell e.s.d.'s are taken into account individually in the estimation of e.s.d.'s in distances, angles and torsion angles; correlations between e.s.d.'s in cell parameters are only used when they are defined by crystal symmetry. An approximate (isotropic) treatment of cell e.s.d.'s is used for estimating e.s.d.'s involving l.s. planes.
Refinement. Refinement of *F*^2^ against ALL reflections. The weighted *R*-factor *wR* andgoodness of fit *S* are based on *F*^2^, conventional *R*-factors *R* are based on *F*, with *F* set to zero for negative *F*^2^. The threshold expression of *F*^2^ > σ(*F*^2^) is used only for calculating *R*-factors(gt) *etc*. and is not relevant to the choice of reflections for refinement. *R*-factors based on *F*^2^ are statistically about twice as large as those based on *F*, and *R*- factors based on ALL data will be even larger. H-atoms attached to carbon were placed in calculated positions (C—H = 0.95 − 0.99 Å) while those attached to nitrogen and oxygen were placed in locations derived from a difference map and their coordinates adjusted to give N—H = 0.91 and O—H = 0.85%A. All were included as riding contributions with isotropic displacement parameters 1.2 − 1.5 times those of the attached atoms. Density associated with an additional lattice water molecule of partial occupancy and disordered about a 2-fold axis was removed with *PLATON* SQUEEZE (Spek, 2015).

### Fractional atomic coordinates and isotropic or equivalent isotropic displacement parameters (Å^2^)

**Table:**

	*x*	*y*	*z*	*U*_iso_*/*U*_eq_	
S1	0.56861 (2)	0.53645 (3)	0.64405 (3)	0.02434 (12)	
O1	0.61317 (4)	0.23972 (11)	0.61342 (10)	0.0415 (3)	
O2	0.35098 (4)	0.52166 (10)	0.63445 (8)	0.0294 (3)	
O3	0.35101 (4)	0.47141 (10)	0.48800 (8)	0.0312 (3)	
N1	0.65244 (4)	0.38651 (11)	0.69843 (9)	0.0247 (3)	
H1A	0.6499	0.4481	0.7318	0.030*	
N2	0.52169 (5)	0.22909 (12)	0.60780 (12)	0.0374 (4)	
H2A	0.5451	0.1805	0.6169	0.045*	
H2B	0.5028	0.2105	0.6410	0.045*	
N3	0.48792 (4)	0.62120 (11)	0.63560 (9)	0.0238 (3)	
C1	0.69789 (5)	0.34988 (13)	0.71843 (11)	0.0236 (3)	
C2	0.71060 (6)	0.26439 (15)	0.66984 (14)	0.0351 (4)	
H2	0.6880	0.2252	0.6207	0.042*	
C3	0.75638 (6)	0.23609 (15)	0.69314 (14)	0.0367 (4)	
H3	0.7646	0.1776	0.6590	0.044*	
C4	0.79040 (6)	0.29031 (15)	0.76441 (13)	0.0326 (4)	
C5	0.77717 (5)	0.37692 (15)	0.81137 (13)	0.0322 (4)	
H5	0.7998	0.4166	0.8599	0.039*	
C6	0.73187 (5)	0.40666 (14)	0.78915 (12)	0.0276 (4)	
H6	0.7238	0.4663	0.8224	0.033*	
C7	0.83973 (6)	0.25687 (19)	0.79060 (16)	0.0463 (5)	
H7A	0.8496	0.2209	0.8537	0.070*	
H7B	0.8585	0.3228	0.7928	0.070*	
H7C	0.8432	0.2052	0.7424	0.070*	
C8	0.61337 (5)	0.33272 (14)	0.64630 (11)	0.0259 (3)	
C9	0.57069 (5)	0.39261 (14)	0.63448 (11)	0.0246 (3)	
C10	0.52928 (5)	0.34191 (14)	0.61994 (11)	0.0262 (3)	
C11	0.49407 (5)	0.42133 (13)	0.61750 (11)	0.0229 (3)	
C12	0.51150 (5)	0.52967 (13)	0.63232 (10)	0.0218 (3)	
C13	0.44362 (5)	0.60840 (13)	0.62383 (11)	0.0238 (3)	
C14	0.41817 (6)	0.71201 (14)	0.62923 (14)	0.0325 (4)	
H14A	0.4140	0.7163	0.6921	0.049*	
H14B	0.3883	0.7111	0.5785	0.049*	
H14C	0.4356	0.7765	0.6206	0.049*	
C15	0.42213 (5)	0.50379 (14)	0.60392 (11)	0.0233 (3)	
C16	0.44682 (5)	0.40837 (13)	0.60086 (11)	0.0235 (3)	
C17	0.42494 (5)	0.29814 (14)	0.57731 (12)	0.0266 (3)	
H17	0.4293	0.2582	0.5257	0.032*	
C18	0.39976 (6)	0.25193 (13)	0.62341 (12)	0.0270 (4)	
H18	0.3956	0.2929	0.6748	0.032*	
C19	0.37747 (5)	0.14278 (13)	0.60252 (12)	0.0261 (3)	
C20	0.38580 (6)	0.06826 (14)	0.53845 (13)	0.0317 (4)	
H20	0.4068	0.0872	0.5068	0.038*	
C21	0.36377 (7)	−0.03324 (15)	0.52028 (14)	0.0384 (4)	
H21	0.3696	−0.0833	0.4761	0.046*	
C22	0.33324 (6)	−0.06161 (15)	0.56647 (15)	0.0409 (5)	
H22	0.3181	−0.1311	0.5538	0.049*	
C23	0.32477 (6)	0.01048 (16)	0.63069 (15)	0.0389 (4)	
H23	0.3039	−0.0093	0.6625	0.047*	
C24	0.34676 (6)	0.11200 (15)	0.64895 (13)	0.0327 (4)	
H24	0.3409	0.1613	0.6936	0.039*	
C25	0.37130 (5)	0.49932 (14)	0.58031 (11)	0.0241 (3)	
C26	0.30137 (6)	0.45925 (16)	0.45613 (13)	0.0360 (4)	
H26A	0.2889	0.4662	0.3853	0.043*	
H26B	0.2882	0.5193	0.4842	0.043*	
C27	0.28784 (6)	0.34965 (16)	0.48538 (13)	0.0353 (4)	
H27A	0.3025	0.2902	0.4611	0.053*	
H27B	0.2546	0.3414	0.4586	0.053*	
H27C	0.2975	0.3456	0.5556	0.053*	
O4	0.52863 (6)	0.82044 (15)	0.59700 (12)	0.0721 (5)	
H4A	0.5180	0.7668	0.6204	0.087*	
H4B	0.5107	0.8246	0.5393	0.087*	

### Atomic displacement parameters (Å^2^)

**Table:**

	*U*^11^	*U*^22^	*U*^33^	*U*^12^	*U*^13^	*U*^23^
S1	0.0185 (2)	0.0275 (2)	0.0272 (2)	−0.00112 (15)	0.00795 (16)	0.00181 (16)
O1	0.0280 (7)	0.0383 (8)	0.0555 (9)	0.0006 (5)	0.0101 (6)	−0.0201 (6)
O2	0.0242 (6)	0.0343 (7)	0.0320 (6)	−0.0004 (5)	0.0125 (5)	0.0014 (5)
O3	0.0201 (6)	0.0462 (8)	0.0243 (6)	−0.0060 (5)	0.0034 (5)	0.0045 (5)
N1	0.0203 (6)	0.0260 (7)	0.0276 (7)	0.0027 (5)	0.0078 (5)	−0.0034 (6)
N2	0.0270 (8)	0.0276 (8)	0.0534 (10)	−0.0002 (6)	0.0077 (7)	−0.0095 (7)
N3	0.0214 (7)	0.0265 (7)	0.0240 (7)	−0.0003 (5)	0.0080 (5)	0.0021 (5)
C1	0.0203 (7)	0.0264 (8)	0.0265 (8)	0.0029 (6)	0.0109 (6)	0.0043 (6)
C2	0.0285 (9)	0.0325 (10)	0.0461 (11)	−0.0018 (7)	0.0148 (8)	−0.0099 (8)
C3	0.0337 (10)	0.0288 (9)	0.0538 (12)	0.0065 (7)	0.0227 (9)	−0.0034 (8)
C4	0.0252 (8)	0.0368 (10)	0.0402 (10)	0.0069 (7)	0.0169 (8)	0.0102 (8)
C5	0.0217 (8)	0.0402 (10)	0.0340 (9)	0.0002 (7)	0.0082 (7)	−0.0002 (8)
C6	0.0261 (8)	0.0300 (9)	0.0283 (9)	0.0026 (7)	0.0113 (7)	−0.0002 (7)
C7	0.0299 (10)	0.0544 (13)	0.0575 (13)	0.0145 (9)	0.0183 (9)	0.0090 (10)
C8	0.0238 (8)	0.0308 (9)	0.0237 (8)	0.0020 (7)	0.0087 (6)	−0.0029 (7)
C9	0.0221 (8)	0.0276 (8)	0.0227 (8)	0.0004 (6)	0.0058 (6)	−0.0032 (6)
C10	0.0232 (8)	0.0271 (8)	0.0260 (8)	0.0008 (6)	0.0050 (6)	−0.0047 (7)
C11	0.0212 (8)	0.0264 (8)	0.0201 (8)	−0.0011 (6)	0.0052 (6)	−0.0012 (6)
C12	0.0189 (7)	0.0295 (9)	0.0166 (7)	−0.0006 (6)	0.0054 (6)	0.0023 (6)
C13	0.0224 (8)	0.0279 (8)	0.0215 (8)	0.0005 (6)	0.0077 (6)	0.0029 (6)
C14	0.0263 (9)	0.0285 (9)	0.0449 (10)	0.0027 (7)	0.0148 (8)	0.0034 (8)
C15	0.0194 (7)	0.0325 (8)	0.0180 (7)	−0.0012 (6)	0.0064 (6)	0.0009 (6)
C16	0.0213 (8)	0.0289 (9)	0.0198 (7)	−0.0038 (6)	0.0060 (6)	−0.0016 (6)
C17	0.0230 (8)	0.0283 (8)	0.0269 (8)	−0.0021 (6)	0.0059 (6)	−0.0049 (7)
C18	0.0278 (8)	0.0261 (9)	0.0269 (8)	−0.0001 (7)	0.0090 (7)	−0.0030 (7)
C19	0.0226 (8)	0.0244 (8)	0.0292 (9)	0.0009 (6)	0.0058 (7)	0.0004 (7)
C20	0.0320 (9)	0.0291 (9)	0.0344 (9)	0.0008 (7)	0.0114 (8)	−0.0011 (7)
C21	0.0416 (11)	0.0247 (9)	0.0439 (11)	0.0019 (8)	0.0076 (9)	−0.0053 (8)
C22	0.0349 (10)	0.0233 (9)	0.0583 (13)	−0.0047 (8)	0.0072 (9)	0.0029 (8)
C23	0.0334 (10)	0.0334 (10)	0.0508 (12)	−0.0021 (8)	0.0152 (9)	0.0087 (9)
C24	0.0316 (9)	0.0305 (9)	0.0385 (10)	−0.0001 (7)	0.0150 (8)	−0.0002 (8)
C25	0.0219 (8)	0.0257 (8)	0.0234 (8)	−0.0009 (6)	0.0058 (6)	0.0056 (6)
C26	0.0211 (8)	0.0486 (11)	0.0317 (9)	−0.0066 (8)	−0.0001 (7)	0.0104 (8)
C27	0.0273 (9)	0.0415 (10)	0.0354 (10)	−0.0079 (8)	0.0081 (8)	0.0008 (8)
O4	0.0651 (11)	0.0779 (12)	0.0635 (11)	−0.0336 (9)	0.0082 (9)	0.0187 (9)

### Geometric parameters (Å, º)

**Table:**

S1—C12	1.7271 (15)	C11—C16	1.415 (2)
S1—C9	1.7459 (17)	C13—C15	1.413 (2)
O1—C8	1.223 (2)	C13—C14	1.497 (2)
O2—C25	1.2030 (19)	C14—H14A	0.9800
O3—C25	1.341 (2)	C14—H14B	0.9800
O3—C26	1.4630 (19)	C14—H14C	0.9800
N1—C8	1.366 (2)	C15—C16	1.394 (2)
N1—C1	1.4145 (19)	C15—C25	1.501 (2)
N1—H1A	0.9101	C16—C17	1.483 (2)
N2—C10	1.384 (2)	C17—C18	1.320 (2)
N2—H2A	0.9102	C17—H17	0.9500
N2—H2B	0.9102	C18—C19	1.473 (2)
N3—C12	1.336 (2)	C18—H18	0.9500
N3—C13	1.3383 (19)	C19—C20	1.391 (2)
C1—C2	1.388 (2)	C19—C24	1.398 (2)
C1—C6	1.394 (2)	C20—C21	1.386 (3)
C2—C3	1.391 (2)	C20—H20	0.9500
C2—H2	0.9500	C21—C22	1.384 (3)
C3—C4	1.383 (3)	C21—H21	0.9500
C3—H3	0.9500	C22—C23	1.376 (3)
C4—C5	1.391 (2)	C22—H22	0.9500
C4—C7	1.506 (2)	C23—C24	1.386 (3)
C5—C6	1.382 (2)	C23—H23	0.9500
C5—H5	0.9500	C24—H24	0.9500
C6—H6	0.9500	C26—C27	1.496 (2)
C7—H7A	0.9800	C26—H26A	0.9900
C7—H7B	0.9800	C26—H26B	0.9900
C7—H7C	0.9800	C27—H27A	0.9800
C8—C9	1.469 (2)	C27—H27B	0.9800
C9—C10	1.376 (2)	C27—H27C	0.9800
C10—C11	1.447 (2)	O4—H4A	0.8502
C11—C12	1.405 (2)	O4—H4B	0.8504
			
C12—S1—C9	90.52 (7)	C13—C14—H14B	109.5
C25—O3—C26	116.17 (13)	H14A—C14—H14B	109.5
C8—N1—C1	127.41 (14)	C13—C14—H14C	109.5
C8—N1—H1A	118.3	H14A—C14—H14C	109.5
C1—N1—H1A	113.7	H14B—C14—H14C	109.5
C10—N2—H2A	121.4	C16—C15—C13	121.24 (14)
C10—N2—H2B	106.6	C16—C15—C25	120.74 (14)
H2A—N2—H2B	113.0	C13—C15—C25	117.92 (14)
C12—N3—C13	116.93 (13)	C15—C16—C11	117.00 (14)
C2—C1—C6	118.58 (14)	C15—C16—C17	122.37 (14)
C2—C1—N1	124.13 (15)	C11—C16—C17	120.58 (14)
C6—C1—N1	117.24 (14)	C18—C17—C16	124.27 (15)
C1—C2—C3	119.92 (17)	C18—C17—H17	117.9
C1—C2—H2	120.0	C16—C17—H17	117.9
C3—C2—H2	120.0	C17—C18—C19	126.06 (15)
C4—C3—C2	122.14 (17)	C17—C18—H18	117.0
C4—C3—H3	118.9	C19—C18—H18	117.0
C2—C3—H3	118.9	C20—C19—C24	118.26 (15)
C3—C4—C5	117.21 (15)	C20—C19—C18	122.69 (15)
C3—C4—C7	121.56 (17)	C24—C19—C18	119.05 (15)
C5—C4—C7	121.23 (17)	C21—C20—C19	120.80 (17)
C6—C5—C4	121.60 (16)	C21—C20—H20	119.6
C6—C5—H5	119.2	C19—C20—H20	119.6
C4—C5—H5	119.2	C22—C21—C20	119.96 (18)
C5—C6—C1	120.53 (16)	C22—C21—H21	120.0
C5—C6—H6	119.7	C20—C21—H21	120.0
C1—C6—H6	119.7	C23—C22—C21	120.21 (17)
C4—C7—H7A	109.5	C23—C22—H22	119.9
C4—C7—H7B	109.5	C21—C22—H22	119.9
H7A—C7—H7B	109.5	C22—C23—C24	119.92 (18)
C4—C7—H7C	109.5	C22—C23—H23	120.0
H7A—C7—H7C	109.5	C24—C23—H23	120.0
H7B—C7—H7C	109.5	C23—C24—C19	120.86 (17)
O1—C8—N1	123.12 (15)	C23—C24—H24	119.6
O1—C8—C9	121.32 (15)	C19—C24—H24	119.6
N1—C8—C9	115.52 (14)	O2—C25—O3	123.99 (14)
C10—C9—C8	124.06 (15)	O2—C25—C15	125.62 (14)
C10—C9—S1	113.42 (12)	O3—C25—C15	110.33 (13)
C8—C9—S1	122.44 (12)	O3—C26—C27	111.33 (14)
C9—C10—N2	124.64 (15)	O3—C26—H26A	109.4
C9—C10—C11	111.68 (14)	C27—C26—H26A	109.4
N2—C10—C11	123.67 (14)	O3—C26—H26B	109.4
C12—C11—C16	117.02 (14)	C27—C26—H26B	109.4
C12—C11—C10	111.35 (13)	H26A—C26—H26B	108.0
C16—C11—C10	131.61 (15)	C26—C27—H27A	109.5
N3—C12—C11	126.03 (14)	C26—C27—H27B	109.5
N3—C12—S1	120.99 (12)	H27A—C27—H27B	109.5
C11—C12—S1	112.96 (11)	C26—C27—H27C	109.5
N3—C13—C15	121.66 (14)	H27A—C27—H27C	109.5
N3—C13—C14	115.83 (14)	H27B—C27—H27C	109.5
C15—C13—C14	122.46 (14)	H4A—O4—H4B	104.0
C13—C14—H14A	109.5		
			
C8—N1—C1—C2	15.6 (3)	C9—S1—C12—C11	−2.44 (12)
C8—N1—C1—C6	−166.89 (15)	C12—N3—C13—C15	−3.2 (2)
C6—C1—C2—C3	0.9 (3)	C12—N3—C13—C14	179.16 (14)
N1—C1—C2—C3	178.41 (16)	N3—C13—C15—C16	3.5 (2)
C1—C2—C3—C4	0.3 (3)	C14—C13—C15—C16	−178.95 (15)
C2—C3—C4—C5	−1.3 (3)	N3—C13—C15—C25	−172.90 (14)
C2—C3—C4—C7	178.42 (18)	C14—C13—C15—C25	4.6 (2)
C3—C4—C5—C6	1.1 (3)	C13—C15—C16—C11	−0.7 (2)
C7—C4—C5—C6	−178.63 (17)	C25—C15—C16—C11	175.66 (13)
C4—C5—C6—C1	0.1 (3)	C13—C15—C16—C17	−178.14 (14)
C2—C1—C6—C5	−1.1 (2)	C25—C15—C16—C17	−1.8 (2)
N1—C1—C6—C5	−178.79 (15)	C12—C11—C16—C15	−2.1 (2)
C1—N1—C8—O1	4.0 (3)	C10—C11—C16—C15	179.78 (16)
C1—N1—C8—C9	−178.01 (14)	C12—C11—C16—C17	175.39 (14)
O1—C8—C9—C10	24.8 (3)	C10—C11—C16—C17	−2.7 (3)
N1—C8—C9—C10	−153.29 (16)	C15—C16—C17—C18	−55.4 (2)
O1—C8—C9—S1	−158.72 (14)	C11—C16—C17—C18	127.21 (18)
N1—C8—C9—S1	23.2 (2)	C16—C17—C18—C19	−179.93 (15)
C12—S1—C9—C10	1.85 (13)	C17—C18—C19—C20	9.1 (3)
C12—S1—C9—C8	−175.01 (14)	C17—C18—C19—C24	−171.14 (17)
C8—C9—C10—N2	−4.8 (3)	C24—C19—C20—C21	0.8 (3)
S1—C9—C10—N2	178.41 (13)	C18—C19—C20—C21	−179.43 (16)
C8—C9—C10—C11	176.00 (14)	C19—C20—C21—C22	−0.3 (3)
S1—C9—C10—C11	−0.79 (18)	C20—C21—C22—C23	−0.2 (3)
C9—C10—C11—C12	−1.04 (19)	C21—C22—C23—C24	0.2 (3)
N2—C10—C11—C12	179.74 (15)	C22—C23—C24—C19	0.3 (3)
C9—C10—C11—C16	177.14 (16)	C20—C19—C24—C23	−0.8 (3)
N2—C10—C11—C16	−2.1 (3)	C18—C19—C24—C23	179.43 (16)
C13—N3—C12—C11	0.1 (2)	C26—O3—C25—O2	−5.3 (2)
C13—N3—C12—S1	178.62 (11)	C26—O3—C25—C15	177.44 (13)
C16—C11—C12—N3	2.6 (2)	C16—C15—C25—O2	117.80 (19)
C10—C11—C12—N3	−178.94 (14)	C13—C15—C25—O2	−65.7 (2)
C16—C11—C12—S1	−176.03 (11)	C16—C15—C25—O3	−64.98 (19)
C10—C11—C12—S1	2.45 (17)	C13—C15—C25—O3	111.48 (16)
C9—S1—C12—N3	178.87 (13)	C25—O3—C26—C27	−79.71 (19)

### Hydrogen-bond geometry (Å, º)

**Table:**

*D*—H···*A*	*D*—H	H···*A*	*D*···*A*	*D*—H···*A*
N1—H1*A*···O2^i^	0.91	2.17	2.9900 (17)	149
N2—H2*A*···O1	0.91	2.25	2.820 (2)	120
O4—H4*A*···N3	0.85	2.04	2.863 (2)	163
O4—H4*B*···N2^ii^	0.85	2.17	2.967 (2)	157

Symmetry codes: (i) −*x*+1, *y*, −*z*+3/2; (ii) −*x*+1, −*y*+1, −*z*+1.
